# Prevalence and genotype distribution of human papillomavirus in cervical cancer and precancer in Japan between 2012 and 2025: a retrospective single-center study

**DOI:** 10.3389/fpubh.2026.1857364

**Published:** 2026-06-12

**Authors:** Hiromi Nakamura, Seiichiro Mori, Yoshiyuki Ishii, Takamasa Takeuchi, Kohsei Tanaka, Airi Imai, Tomoya Matsui, Takashi Iwata, Wataru Yamagami, Iwao Kukimoto

**Affiliations:** 1 Pathogen Genomics Center, National Institute of Infectious Diseases, Japan Institute for Health Security, Tokyo, Japan; 2 Department of Obstetrics and Gynecology, Keio University School of Medicine, Tokyo, Japan

**Keywords:** adenocarcinoma, genotype distribution, human papillomavirus, prevalence ratio, squamous cell carcinoma, vaccination

## Abstract

**Objective:**

This study aimed to provide the latest data on the distribution of human papillomavirus (HPV) genotypes in cervical cancer and precancer at a single university hospital in Tokyo, Japan, between 2012 and 2025.

**Methods:**

Cervical exfoliated cell specimens collected from 2,536 women with histologically confirmed, cervical intraepithelial neoplasia 2 (CIN2), CIN3, squamous cell carcinoma (SCC), adenocarcinoma *in situ* (AIS), adenosquamous cell carcinoma (ASC), and adenocarcinoma (ADC), were tested by HPV L1 PCR followed by reverse blot hybridization for HPV genotyping.

**Results:**

Overall, HPV positivity was 96.6%, with high detection rates in CIN2 (98.5%), CIN3 (98.2%), and SCC (96.1%), and slightly lower rates in ADC (78.3%). In squamous lesions, HPV16 infections were the most prevalent and increased progressively with lesion severity (33.2% in CIN2, 50.1% in CIN3, 61.7% in SCC). Prevalence ratio analysis demonstrated a significantly higher prevalence of HPV16 in CIN3 versus CIN2 and in SCC versus CIN3, supporting its strong association with progression to invasive cancer. In contrast, HPV52 and HPV58 infections decreased with disease progression. In glandular lesions, HPV16 and HPV18 predominated across AIS, ASC, and ADC. Among ADC subtypes, usual-type ADC showed the highest HPV positivity (89.8%), whereas endometrioid, minimal deviation, serous, and clear-cell subtypes showed low or absent HPV positivity, highlighting the etiologic heterogeneity of ADC. Among single-type infection cases, the proportion of lesions attributable to vaccine-preventable types (HPV16/18) increased with severity in squamous lesions (31.9% in CIN2 to 72.2% in SCC) and remained consistently high in glandular lesions (>94%). Regarding HPV16/18/31/33/45/52/58, potential coverage exceeded 87% in CIN2 and 92% in SCC.

**Conclusion:**

These findings confirm the central role of HPV16 in cervical carcinogenesis, and highlight the contribution of HPV52 and HPV58 in precancerous lesions, which further underscores the need for the widespread adoption of the nonavalent vaccine in Japan.

## 1 Introduction

Cervical cancer ranks fourth worldwide in both cancer morbidity and mortality, primarily caused by persistent infection with oncogenic human papillomavirus (HPV) (1). Of more than 400 HPV genotypes identified to date (The Papillomavirus Episteme, https://pave.niaid.nih.gov/), about 15 types (HPV16, 18, 31, 33, 35, 39, 45, 51, 52, 56, 58, 59, 68, 73, and 82) are defined as “high-risk” types (2), because they are frequently detected in invasive cervical cancer and its precancerous lesions (3, 4). Worldwide, approximately 70% of cervical cancers are attributable to HPV16 and HPV18 (5), and they are the primary targets for vaccination aimed at reducing cervical cancer incidence. The bivalent (HPV16/18) and quadrivalent (HPV6/11/16/18) vaccines both target HPV16/18, while the nonavalent (HPV6/11/16/18/31/33/45/52/58) vaccine includes an additional five high-risk types. The prevalence of the high-risk HPV types other than HPV16/18 varies by region and country (6), necessitating tailored strategies for vaccination and cervical screening in each area.

Moreover, the distribution of HPV genotypes also differs according to the histological subtype of cervical cancer and its precursor lesions. Invasive cervical cancer is broadly classified into squamous cell carcinoma (SCC) and adenocarcinoma (ADC) based on the squamous and glandular tissue origins of the cancerous cells, respectively. These cancers develop through their precursor lesions: cervical intraepithelial neoplasia 2 (CIN2) and CIN3 progress to SCC and adenocarcinoma *in situ* (AIS) progresses to ADC. In addition, adenosquamous cell carcinoma (ASC) is a distinct malignant tumor defined by the coexistence of both ADC and SCC components, each of which is unequivocally malignant on histologic examination. Different HPV type distributions observed among cervical cancers and precancers reflect different tissue tropisms of high-risk HPVs and their different risks for cancer progression.

When considering strategies for cervical cancer prevention, it is crucial to monitor the distribution of HPV genotypes in individual regions and countries. To estimate the potential impact of vaccination on cervical cancer incidence in Japan, we previously reported baseline data on HPV type distribution among Japanese women with cervical cancer and precancer in the pre-vaccination era and assessed relative risks of individual HPV types for cancer progression (7). In the current study, we aim to update the epidemiological information on HPV type distribution among cervical malignancies in Japan. We therefore conducted a retrospective analysis of the data on cervical specimens collected continuously at a single university hospital between 2012 and 2025.

## 2 Materials and methods

### 2.1 Participant selection and specimen collection

**Figure 1:**
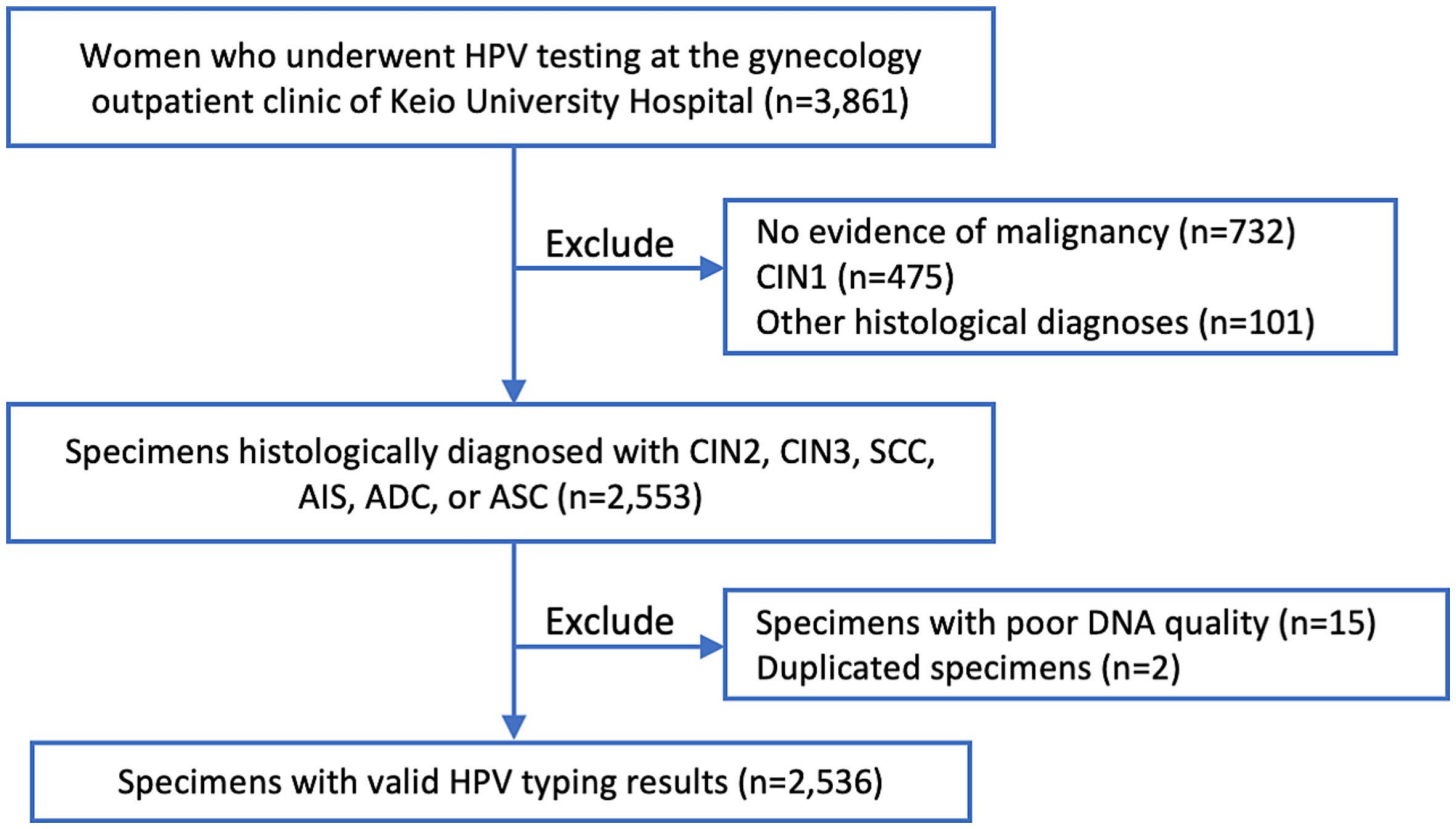
Flow chart of specimen selection.

We conducted a retrospective study at Keio University Hospital in Tokyo covering the period from January 2012 to December 2025. The criteria to participate in the study were as follows: (1) female sex, (2) were aged ≥18 years, (3) were negative for HIV infection, (4) were histologically diagnosed with CIN2, CIN3, SCC, AIS (includes AIS and AIS + any CIN), ADC, or ASC by punch biopsy or cervical conization, (5) had valid HPV typing results. Histological diagnosis was made by experienced pathologists using hematoxylin/eosin-stained sections according to the World Health Organization classification. When diagnoses between punch biopsy and cervical conization were discordant, the higher-grade lesion was consistently adopted as the final diagnosis for analysis, regardless of whether the higher-grade diagnosis was identified in the punch biopsy specimen or in the conization specimen (e.g., if a punch biopsy was CIN2 and a cone biopsy was CIN3, the CIN3 diagnosis was used). The flow chart of specimen selection is presented in Figure 1. Exfoliated ecto- and endo-cervical cells were collected before colposcopy using Cervex-Brush^®^ Combi (Rovers Medical Devices B. V., The Netherlands) and stored in ThinPrep PreservCyt solution (Hologic, Bedford, MA, USA) at 4 °C until DNA extraction. All participants entered the study after providing voluntary, informed consent.

### 2.2 HPV genotyping

Total cellular DNA was extracted from cervical exfoliated cells using the MagNA Pure LC Total Nucleic Acid Isolation kit (Roche, Indianapolis, IN, USA). The purified DNA was PCR-amplified with AmpliTaq Gold polymerase (Thermo Fisher Scientific, Waltham, MA, USA) and biotinylated PGMY09/11 primers to amplify the L1 gene of mucosal HPVs. Biotinylated human leukocyte antigen (HLA) primers were used to amplify cellular HLA DNA. Positive (0.1 pg/mL of HPV16 full-length genomic DNA in a plasmid) and negative (dH_2_O) controls were used to assess the sensitivity of PCR and to detect contaminating HPV DNA in reagents. The PCR products were analyzed on 1.5% agarose gels to examine amplification of HPV and HLA DNA. HLA DNA amplification served as an internal control to confirm the integrity of the extracted DNA, and the samples that failed to amplify HLA DNA were excluded due to poor DNA quality. Reverse blotting hybridization was performed as previously described (7). Briefly, denatured PCR products were allowed to hybridize with oligonucleotide probes specific for 31 HPV genotypes (HPV6, 11, 16, 18, 26, 31, 33, 34, 35, 39, 40, 42, 44, 45, 51, 52, 53, 54, 55, 56, 57, 58, 59, 66, 68, 69, 70, 73, 82, 83, and 84) immobilized on a Biodyne C membrane (Pall corporation, Port Washington, NY, USA) using a Miniblotter MN45 (Immunetics, Cambridge, MA, USA). The hybridized DNA was detected using horseradish peroxidase-conjugated streptavidin (Cytiva, Little Chalfont, UK) and the ECL detection reagents (Cytiva).

### 2.3 Statistical analysis

A generalized linear model with a binomial distribution and a log link was used to estimate the prevalence ratio (PR) of high-risk HPVs across histological grades, with 95% confidence intervals (CIs). The PR was adjusted based on the women’s age at diagnosis. All statistical analyses were performed using R version 4.2.3.

## 3 Results

### 3.1 Overall prevalence of HPV infection

**Figure 2:**
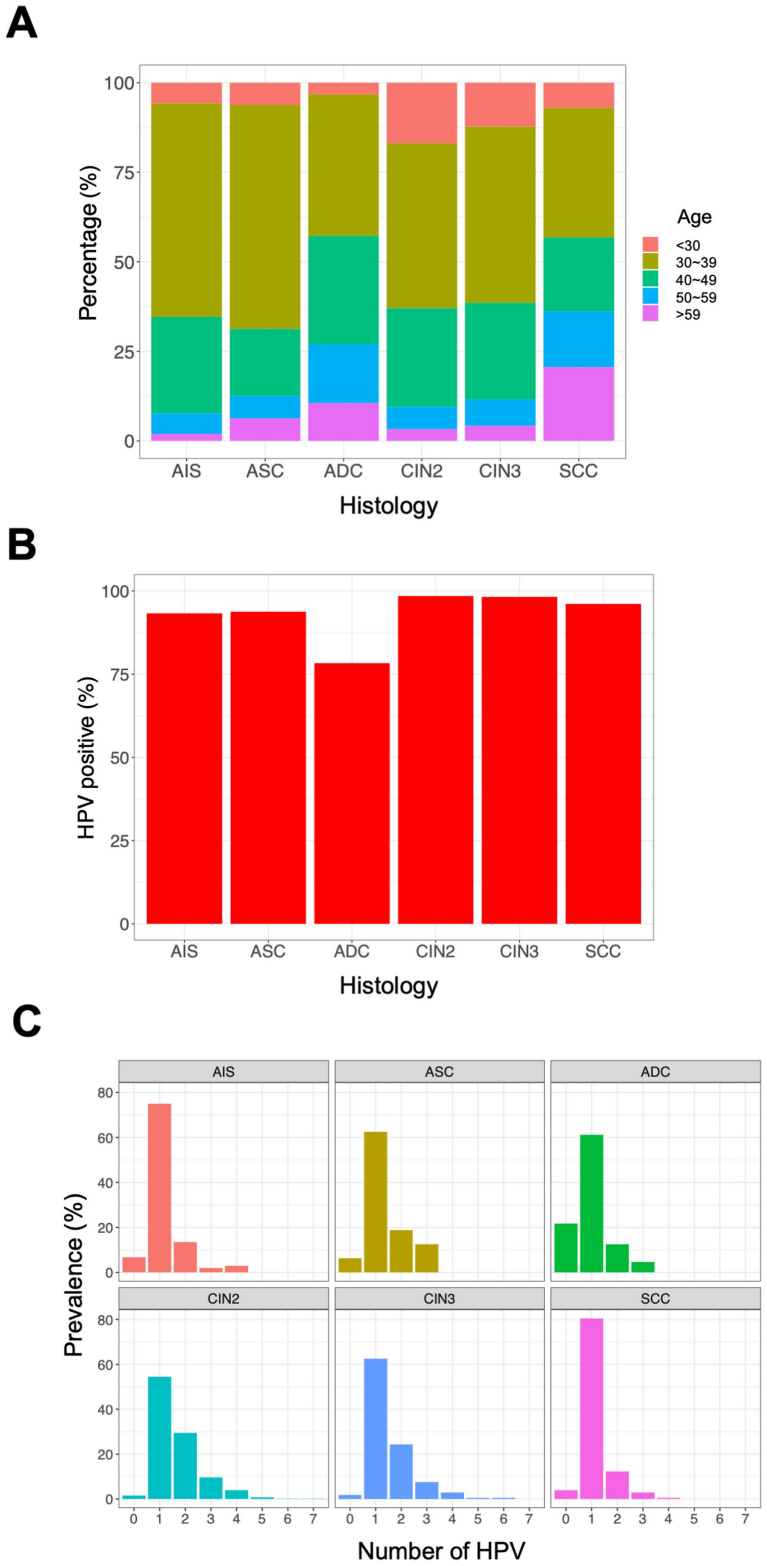
Human papillomavirus infection status among women with cervical lesions in Japan. **(A)** Age distribution of study participants by histology. **(B)** Positivity of HPV infection by histology. **(C)** Number of HPV types detected in individual specimens. AIS, adenocarcinoma *in situ*; ASC, adenosquamous cell carcinoma; ADC, adenocarcinoma; CIN2, cervical intraepithelial neoplasia grade 2; CIN3, cervical intraepithelial neoplasia grade 3; SCC, squamous cell carcinoma.

A total of 2,536 cervical swab samples (CIN2, *n* = 1,042; CIN3, *n* = 838; SCC, *n* = 384; AIS, *n* = 104; ASC, *n* = 16; ADC, *n* = 152) were analyzed in this study (Tables 1, 2). Age distributions in each category (average age ± standard deviation) were as follows: CIN2 (37.9 ± 9.5 years), CIN3 (38.6 ± 9.7 years), SCC (46.3 ± 14.7 years), AIS (38.3 ± 7.7 years), ASC (39.1 ± 8.7 years), and ADC (44.2 ± 11.6 years). When stratified by age group, cancer specimens (ASC, ADC, and SCC) were more frequently collected from older women (aged 60 years and older) than precancerous specimens (Figure 2A). Overall, 2,449 cases (96.6%) were HPV-positive: CIN2, *n* = 1,026 (98.5%); CIN3, *n* = 823 (98.2%); SCC, *n* = 369 (96.1%); AIS, *n* = 97 (93.3%); ASC, *n* = 15 (93.8%); ADC, *n* = 119 (78.3%) (Figure 2B). Among the HPV-positive cases, infection with a single HPV type was detected in 1,582 cases (1,582/2,449, 64.6%): CIN2, *n* = 568 (568/1,026, 55.4%); CIN3, *n* = 524 (524/823, 63.7%); SCC, *n* = 309 (309/369, 83.7%); AIS, *n* = 78 (78/97, 80.4%); ASC, *n* = 10 (10/15, 66.7%); ADC, *n* = 93 (93/119, 78.2%) (Figure 2C). Multiple HPV infections were detected in 867 cases (867/2,449, 35.4%): CIN2, *n* = 458 (458/1,026, 44.6%); CIN3, *n* = 299 (299/823, 36.3%); SCC, *n* = 60 (60/369, 16.3%); AIS, *n* = 19 (19/97, 19.6%); ASC, *n* = 5 (5/15, 33.3%); ADC, *n* = 26 (26/119, 21.8%). The maximum number of HPV types in multiple infections was 7 in CIN2.

**Table 1:** Human papillomavirus status by cervical squamous histology.

Histology	CIN2	CIN3	SCC
*N*	1,042	838	384
Median age	36	37	42
Mean age ± SD	37.9 ± 9.5	38.6 ± 9.7	46.3 ± 14.7
Age range	19–87	18–81	22–86
HPV positive (%)	1,026 (98.5)	823 (98.2)	369 (96.1)
HPV negative (%)	16 (1.5)	15 (1.8)	15 (3.9)
Single infections (%)^*^	568 (55.4)	524 (63.7)	309 (83.7)
HPV16 (%)^**^	166 (29.2)	246 (46.9)	196 (63.4)
HPV18 (%)^**^	15 (2.6)	12 (2.3)	27 (8.7)
HPV31 (%)^**^	65 (11.4)	54 (10.3)	9 (2.9)
HPV33 (%)^**^	24 (4.2)	16 (3.1)	4 (1.3)
HPV35 (%)^**^	5 (0.9)	9 (1.7)	4 (1.3)
HPV39 (%)^**^	7 (1.2)	5 (1.0)	4 (1.3)
HPV45 (%)^**^	3 (0.5)	2 (0.4)	4 (1.3)
HPV51 (%)^**^	16 (2.8)	9 (1.7)	1 (0.3)
HPV52 (%)^**^	134 (23.6)	82 (15.6)	27 (8.7)
HPV56 (%)^**^	5 (0.9)	5 (1.0)	3 (1.0)
HPV58 (%)^**^	88 (15.5)	56 (10.7)	19 (6.1)
HPV59 (%)^**^	3 (0.5)	2 (0.4)	1 (0.3)
HPV68 (%)^**^	4 (0.7)	2 (0.4)	2 (0.6)
HPV73 (%)^**^	1 (0.2)	0 (0.0)	0 (0.0)
HPV82 (%)^**^	8 (1.4)	14 (2.7)	4 (1.3)
Other (%)^**^	24 (4.2)	10 (1.9)	4 (1.3)
Multiple infections (%)^*^	458 (44.6)	299 (36.3)	60 (16.3)

^*^Percentage of HPV positive; ^**^Percentage of single infections. CIN2, cervical intraepithelial neoplasia grade 2; CIN3, cervical intraepithelial neoplasia grade 3; SCC, squamous cell carcinoma; SD, standard deviation.

**Table 2:** Human papillomavirus status by cervical glandular histology.

Histology	AIS	ASC	ADC total	ADC by subtype				
				Usual-type	Endometrioid	Minimal deviation	Serous	Clear-cell
*N* (%)^*^	104	16	152	128 (84.2)	11 (7.2)	10 (6.6)	2 (1.3)	1 (0.7)
Median age	36	37.5	41	40	59	47.5	41.5	56
Mean age ± SD	38.3 ± 7.7	39.1 ± 8.7	44.2 ± 11.6	42.7 ± 10.8	58.3 ± 14.7	47.1 ± 9.0	41.5	56
Age range	24–62	26–62	22–78	22–77	37–78	33–62	37–46	56
HPV positive (%)	97 (93.3)	15 (93.8)	119 (78.3)	115 (89.8)	2 (18.2)	2 (20.0)	0 (0.0)	0 (0.0)
HPV negative (%)	7 (6.7)	1 (6.3)	33 (21.7)	13 (9.4)	9 (81.8)	8 (80.0)	2 (100)	1 (100)
Single infections (%)^**^	78 (80.4)	10 (66.7)	93 (78.2)	90 (78.3)	2 (100)	1 (50.0)	NA	NA
HPV16 (%)^***^	32 (41.0)	5 (50.0)	45 (48.4)	44 (48.9)	1 (50.0)	–	NA	NA
HPV18 (%)^***^	41 (52.6)	5 (50.0)	43 (46.2)	41 (45.6)	1 (50.0)	1 (100)	NA	NA
HPV45 (%)^***^	2 (2.6)	–	1 (1.1)	1 (1.1)	–	–	NA	NA
HPV51 (%)^***^	1 (1.3)	–	–	–	–	–	NA	NA
HPV52 (%)^***^	–	–	2 (2.2)	2 (2.2)	–	–	NA	NA
HPV54 (%)^***^	–	–	1 (1.1)	1 (1.1)	–	–	NA	NA
HPV59 (%)^***^	1 (1.3)	–	1 (1.1)	1 (1.1)	–	–	NA	NA
HPV62^****^ (%)^***^	1 (1.3)	–	–	–	–	–	NA	NA
Multiple infections (%)^**^	19 (19.6)	5 (33.3)	26 (21.8)	25 (21.7)	0 (0.0)	1 (50.0)	NA	NA

^*^Percentage of all ADC; ^**^Percentage of HPV positive; ^***^Percentage of single infections. ^****^HPV62 was determined by sequencing of HPV PCR product.

AIS, adenocarcinoma in situ (includes AIS and AIS + any cervical intraepithelial neoplasia); ASC, adenosquamous cell carcinoma; ADC, adenocarcinoma; NA, not applicable; SD, standard deviation.

### 3.2 HPV genotype prevalence in cervical squamous lesions

**Figure 3:**
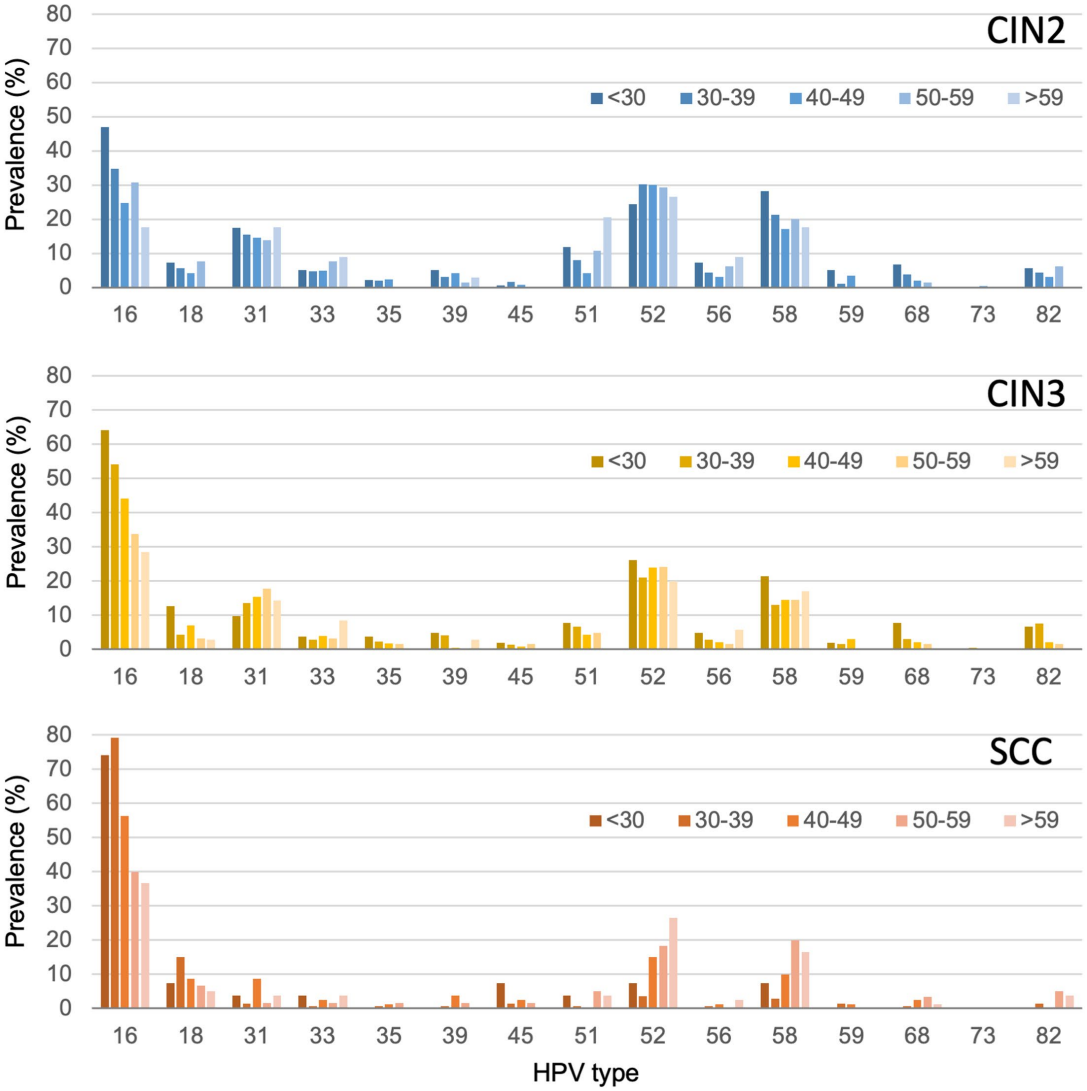
Prevalence of 15 high-risk HPV genotype in cervical squamous lesions by age group. CIN2, cervical intraepithelial neoplasia grade 2; CIN3, cervical intraepithelial neoplasia grade 3; SCC, squamous cell carcinoma.

The distribution of individual HPV types was analyzed according to histological type in cervical squamous lesions. Overall, the five most common types in each histology were as follows: in CIN2, HPV16 (33.2%), HPV52 (29.0%), HPV58 (21.1%), HPV31 (15.5%), HPV51 (8.2%); in CIN3, HPV16 (50.1%), HPV52 (22.7%), HPV58 (14.8%), HPV31 (14.0%), HPV18 (6.0%); in SCC, HPV16 (61.7%), HPV52 (10.9%), HPV18 (10.2%), HPV58 (8.3%), HPV31 (4.7%). When analyzed by age group, nearly identical distribution patterns were observed across all age groups in each histological type (Figure 3), although HPV16 prevalence decreased with age in all histological types. In contrast, the prevalence of HPV52 and HPV58 increased in women aged 40 years and older in SCC. When restricted to cases of single infections, the five most frequent types were as follows: in CIN2, HPV16 (29.2%), HPV52 (23.6%), HPV58 (15.5%), HPV31 (11.4%), HPV33 (4.2%); in CIN3, HPV16 (46.9%), HPV52 (15.6%), HPV58 (10.7%), HPV31 (10.3%), HPV33 (3.1%); in SCC, HPV16 (63.4%), HPV52 (8.7%), HPV18 (8.7%), HPV58 (6.1%), HPV31 (2.9%). Because multiple infections were frequently observed in CIN2 and CIN3, a hierarchical attribution method was used to estimate the prevalence of the genotypes responsible for these lesions. For example, using the hierarchical method, a specimen containing HPV16, HPV31, and HPV52 was assigned exclusively to HPV16, which was the most prevalent (“hierarchical”) genotype detected in the CIN2/3 samples. As shown in Supplementary Figure 1, the prevalence of HPV16 and HPV52 in multiple infection cases was slightly increased compared to that in single infection cases, while that of HPV31 was slightly decreased. When single and multiple infections were estimated together, the results showed a trend nearly identical to that of single infections alone.

**Figure 4:**
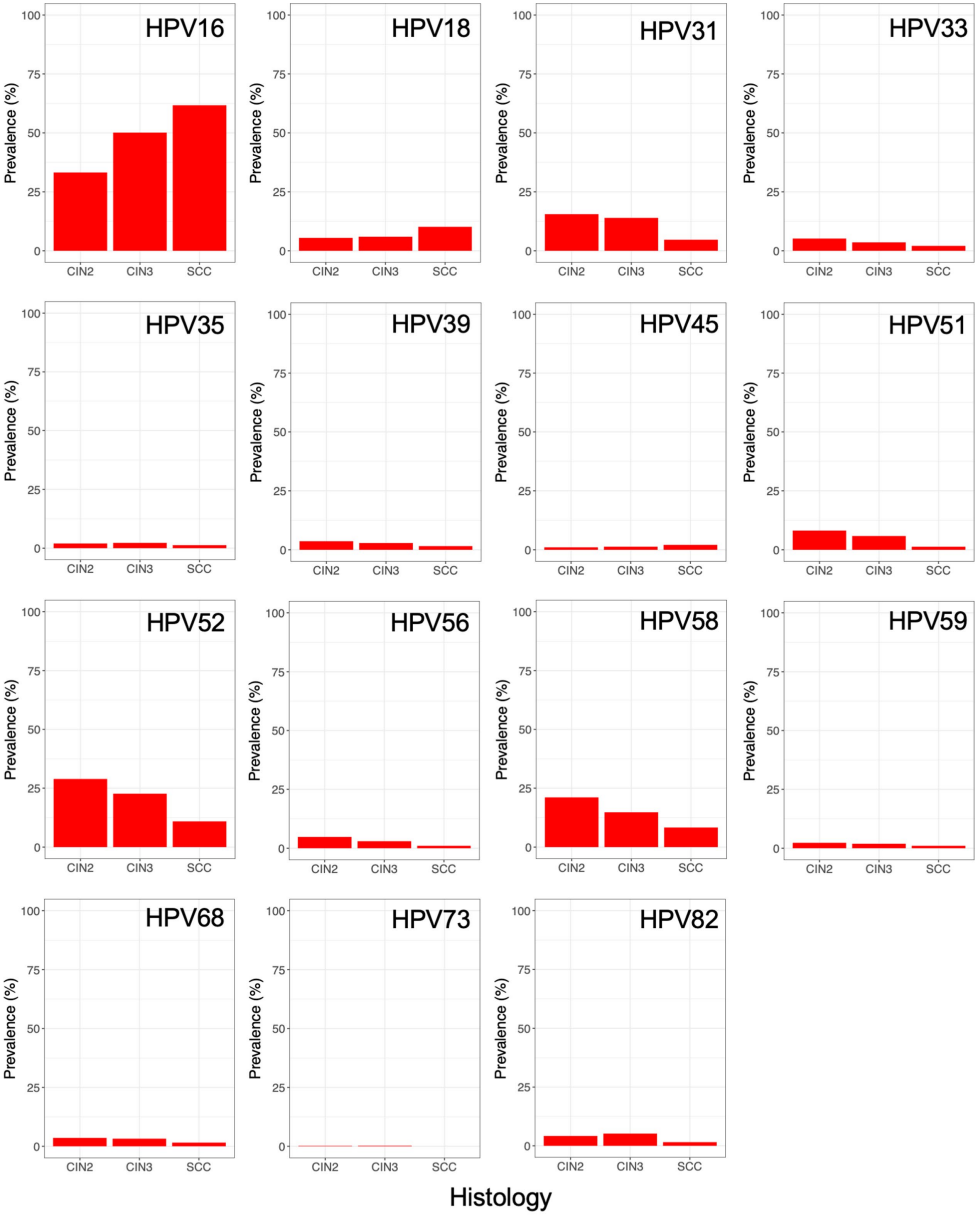
Prevalence of 15 high-risk HPV genotypes throughout cervical squamous lesions. CIN2, cervical intraepithelial neoplasia grade 2; CIN3, cervical intraepithelial neoplasia grade 3; SCC, squamous cell carcinoma.

When the detection rates of 15 high-risk types were compared among CIN2, CIN3, and SCC, the prevalence of HPV16 increased progressively from CIN2 to SCC, while the prevalence of HPV18 and HPV45 remained stable in CIN2 and CIN3 but increased from CIN3 to SCC (Figure 4). In contrast, the prevalence of HPV31, HPV33, HPV39, HPV51, HPV52, HPV56, HPV58, HPV59, and HPV68 progressively decreased from CIN2 to SCC. The prevalence of HPV82 was higher in CIN3 than in CIN2 but lower in SCC than in CIN2.

To evaluate the relative risk of progression to invasive cervical cancer by individual HPV types, the prevalence ratio (PR) of 15 high-risk types was calculated by comparing the detection rates between CIN2, CIN3, and SCC. As shown in Table 3, HPV16 prevalence was significantly higher in CIN3 compared with CIN2 (PR = 1.52, 95% CI = 1.37–1.70). Conversely, the prevalence of HPV52, HPV56, and HPV58 significantly decreased from CIN2 to CIN3 (HPV52, PR = 0.78, 95% CI = 0.67–0.91; HPV56, PR = 0.63, 95% CI = 0.38–0.99; HPV58, PR = 0.71, 95% CI = 0.58–0.86). HPV16 showed an even higher prevalence in SCC than CIN3 (PR = 1.39, 95% CI = 1.26–1.52), and HPV18 also exhibited significantly higher prevalence (PR = 1.98, 95% CI = 1.31–2.97). In contrast, the prevalence of HPV31, HPV51, HPV52, HPV56, HPV58, and HPV82 was significantly decreased in SCC compared with CIN3.

**Table 3:** Prevalence ratios of HPV genotypes between different histology.

Type	CIN3 vs. CIN2 (95% CI)	SCC vs. CIN3 (95% CI)
HPV16	**1.52 (1.37–1.70)**	**1.39 (1.26–1.52)**
HPV18	1.11 (0.77–1.60)	**1.98 (1.31–2.97)**
HPV31	0.90 (0.72–1.12)	**0.31 (0.18–0.50)**
HPV33	0.68 (0.43–1.04)	0.50 (0.21–1.07)
HPV35	1.15 (0.62–2.13)	0.63 (0.20–1.60)
HPV39	0.80 (0.48–1.32)	0.65 (0.24–1.49)
HPV45	1.28 (0.55–2.98)	1.94 (0.74–4.83)
HPV51	0.73 (0.51–1.01)	**0.25 (0.09–0.58)**
HPV52	**0.78 (0.67–0.91)**	**0.45 (0.32–0.61)**
HPV56	**0.63 (0.38–0.99)**	**0.32 (0.09–0.86)**
HPV58	**0.71 (0.58–0.86)**	**0.52 (0.35–0.76)**
HPV59	0.85 (0.44–1.57)	0.59 (0.17–1.66)
HPV68	0.96 (0.58–1.55)	0.59 (0.22–1.35)
HPV73	1.26 (0.15–10.5)	ND
HPV82	1.29 (0.86–1.94)	**0.36 (0.14–0.77)**

CIN2, cervical intraepithelial neoplasia grade 2; CIN3, cervical intraepithelial neoplasia grade 3; SCC, squamous cell carcinoma; PR, prevalence ratio; CI, confidence interval.

Statistically significant values are bolded. ND, not determined.

### 3.3 HPV genotype prevalence in cervical glandular lesions

**Figure 5:**
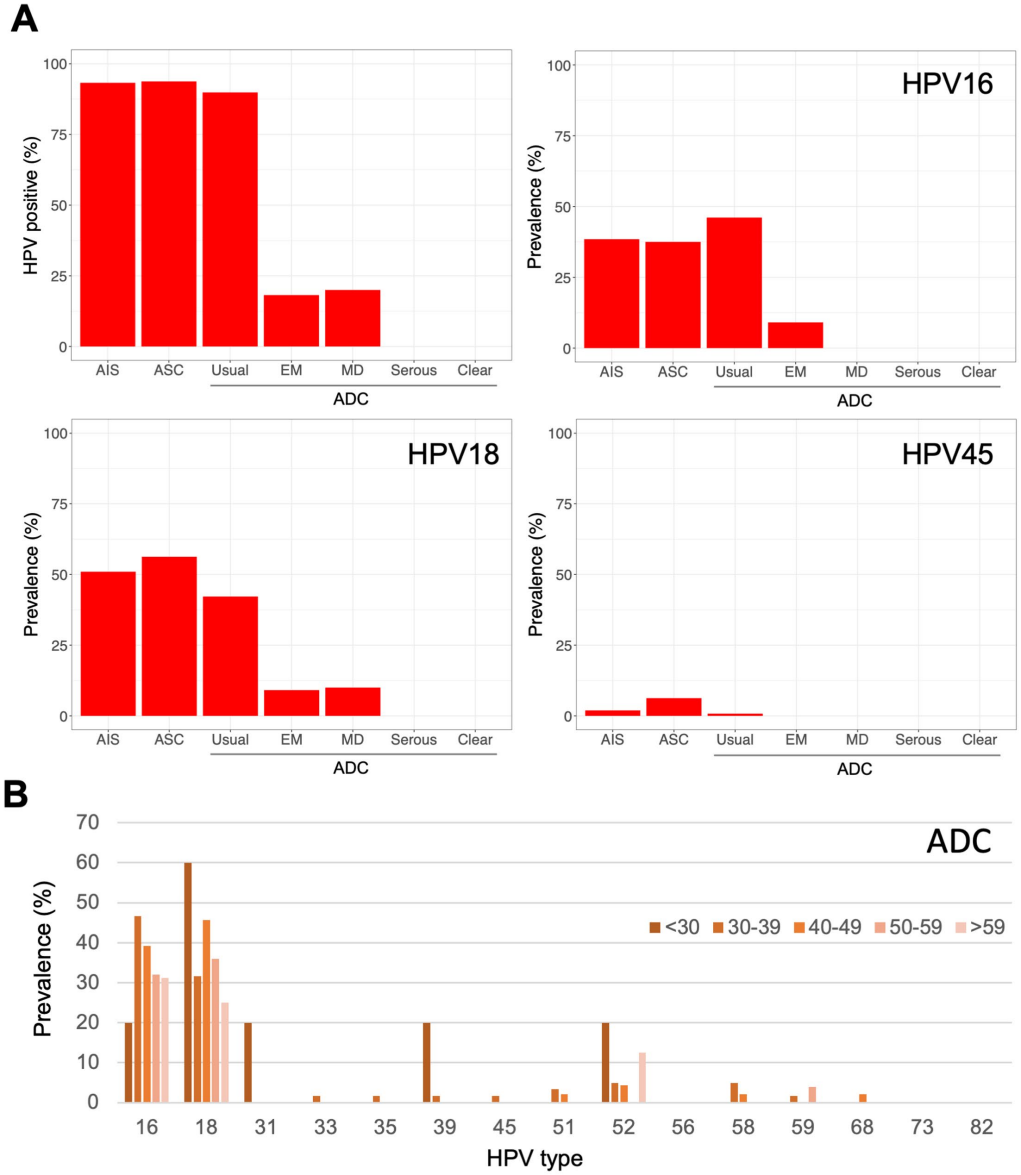
Prevalence of HPV genotypes in cervical glandular lesions. **(A)** Prevalence of any HPV, HPV16, HPV18, and HPV45. **(B)** Prevalence of 15 high-risk HPV genotypes in cervical adenocarcinoma by age group. AIS, adenocarcinoma *in situ*; ASC, adenosquamous cell carcinoma; ADC, adenocarcinoma; Usual, usual-type ADC; EM, endometrioid ADC; MD, minimal deviation ADC; Serous, serous ADC; Clear, clear-cell ADC.

Overall, HPV16 and HPV18 were the most prevalent across all glandular lesions (Figure 5A): in AIS, HPV16 (38.5%) and HPV18 (51.0%); in ASC, HPV16 (37.5%) and HPV18 (56.2%); in ADC, HPV16 (39.5%) and HPV18 (36.8%). When restricted to single infections, the detection rates of HPV16 and HPV18 were as follows: in AIS, HPV16 (41.0%) and HPV18 (52.6%); in ASC, HPV16 (50.0%) and HPV18 (50.0%); in ADC, HPV16 (48.4%) and HPV18 (46.2%). Even when analyzed by age group, HPV16 and HPV18 were predominant across all age groups in ADC (Figure 5B).

Since cervical ADC encompasses diverse histological subtypes (i.e., usual-type, endometrioid, minimal deviation, serous, and clear-cell ADC), a more detailed examination was conducted for each histological subtype. Among the ADC cases (*n* = 152), there were 128 cases of usual-type ADC (84.2%), 11 cases of endometrioid ADC (7.2%), 10 cases of minimal deviation ADC (6.6%), 2 cases of serous ADC (1.3%), and 1 case of clear-cell ADC (0.7%). The average age of each histological subtype was as follows: usual-type ADC (42.7 years), endometrioid ADC (58.3 years), minimal deviation ADC (47.1 years), serous ADC (41.5 years), and clear-cell ADC (56 years). As shown in Table 2, HPV positivity varied significantly across tumor subtypes. Usual-type ADC showed HPV positivity of 89.8%, while other subtypes showed low prevalence or absence of HPV: endometrioid ADC (18.2%), minimal deviation ADC (20.0%), serous ADC (0%), and clear-cell ADC (0%). When restricted to single infections, the prevalence of HPV16 and HPV18 was as follows: in usual-type ADC, HPV16 (48.9%) and HPV18 (45.6%); in endometrioid ADC, HPV16 (50.0%) and HPV18 (50.0%); in minimal deviation ADC, HPV16 (100%).

### 3.4 Prevalence of vaccine-preventable genotypes in HPV-positive cervical cancer and precancer

**Figure 6:**
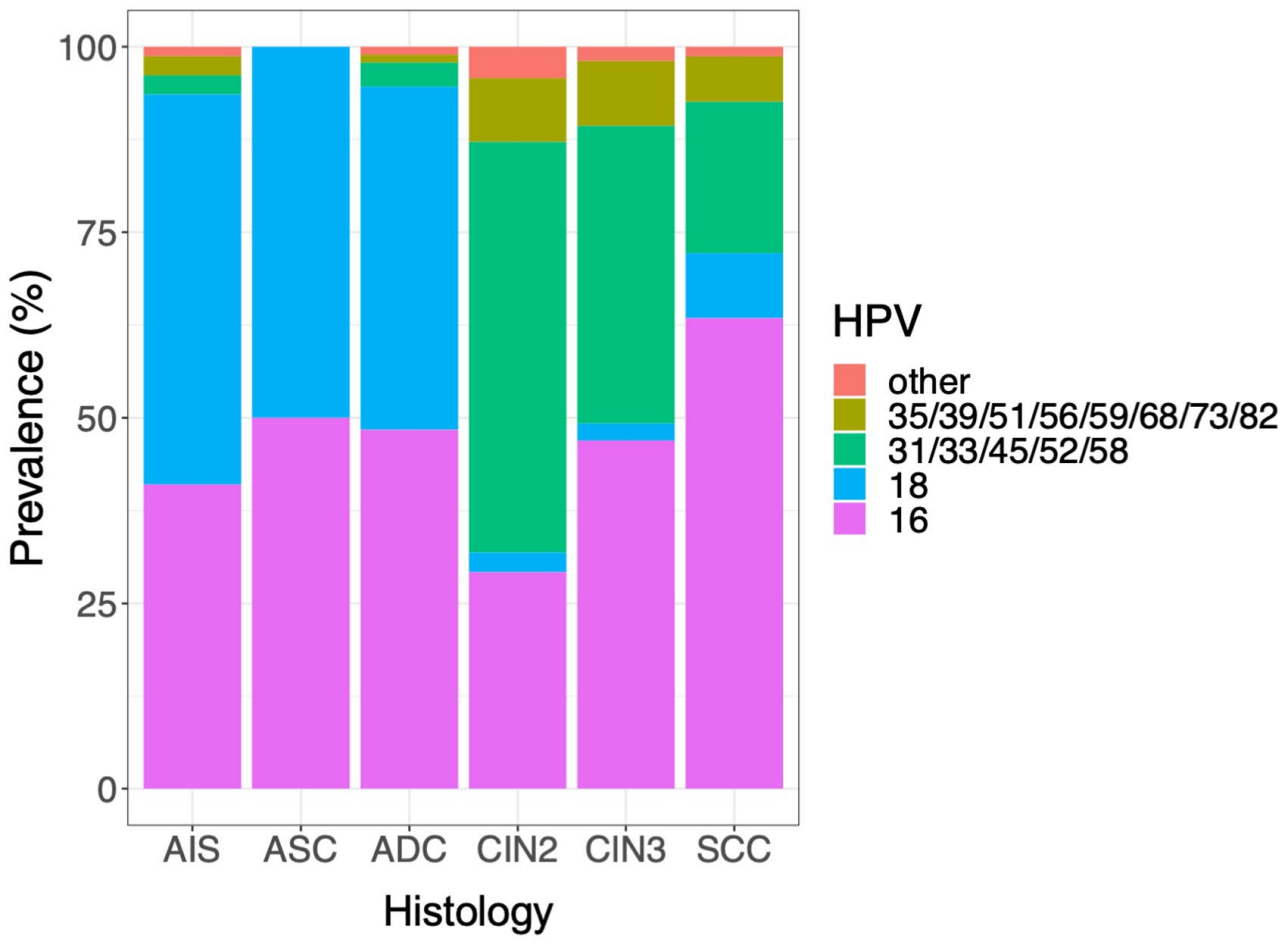
Prevalence of HPV genotypes included or not included in HPV vaccines. AIS, Adenocarcinoma *in situ*; ASC, adenosquamous cell carcinoma; ADC, adenocarcinoma; CIN2, cervical intraepithelial neoplasia grade 2; CIN3, cervical intraepithelial neoplasia grade 3; SCC, squamous cell carcinoma.

Based on the distribution of HPV types in single-infection cases, the impact of the bivalent/quadrivalent or nonavalent vaccine on preventing HPV-positive cervical precancer and cancer was estimated. As shown in Figure 6, among squamous lesions, the combined prevalence of HPV16/18 significantly increased from CIN2 to SCC: in CIN2, 31.9% (95% CI = 28.0–35.7%); in CIN3, 49.2% (95% CI = 45.0–53.5%); in SCC, 72.2% (95% CI = 67.2–77.2%). In contrast, among glandular lesions, HPV16/18 prevalence was constantly high from AIS to ADC: in AIS, 93.6% (95% CI = 88.2–99.0%); in ASC, 100%; in ADC (95% CI, not determined), 94.6% (95% CI = 90.0–99.2%). Regarding HPV16/18/31/33/45/52/58, the overall positivity was further increased: in CIN2, 87.1% (95% CI = 84.4–89.9%); in CIN3, 89.3% (95% CI = 86.7–92.0%); in SCC, 92.6% (95% CI = 89.6–95.5%); in AIS, 96.2% (95% CI = 91.9–100.4%); in ASC, 100% (95% CI, not determined); in ADC, 97.8% (95% CI = 94.9–100.8%). When considering both HPV-positive and -negative cervical cancers, the estimated coverage with the bivalent/quadrivalent vaccines was 69.4% in SCC and 74.1% in ADC, and with the nonavalent vaccine was 89.0% in SCC and 76.6% in ADC. These estimates were almost consistent with those reported in a recent meta-analysis (bivalent/quadrivalent vaccine types: 68.7% in SCC and 72.1% in ADC; nonavalent vaccine types: 76.5% in SCC and 74.3% in ADC) (8).

## 4 Discussion

This study reports the latest data on HPV genotype distribution among women diagnosed with cervical cancer and precancerous lesions in Japan. Cervical swab specimens were collected from 2012 to 2025 at a single university hospital in Tokyo, Japan’s largest capital city, and were retrospectively analyzed for HPV genotype prevalence. In cervical squamous lesions, HPV16 was the predominant type throughout CIN2, CIN3, and SCC, followed by HPV52, HPV58, and HPV31. In contrast, HPV16 and HPV18 were the two predominant types in cervical glandular lesions. These distribution patterns are very similar to those of previous studies (9–12) and the meta-analysis of HPV type prevalence in Japan (8).

Recently, Onuki et al. (12) analyzed HPV typing data from 5,045 Japanese women diagnosed with cervical cancer and precancer; however, that study was limited to women under 40 years of age. In contrast, our study includes women across a wider age range, from 18 to 87 years. Interestingly, in older age groups (over 50s), the HPV16 detection rate was lower in CIN3 and SCC when compared to younger age groups, while infections with HPV52 and HPV58 increased. Since most HPV infections occur in young adults in their 20s and 30s, these results may suggest a different natural history between HPV16 and HPV52/58; HPV16 infection progresses rapidly to SCC, whereas HPV52/58 infection takes a long time to progress to cancer. The reason for this difference remains unclear, but it may be due to subtle differences in the viral life cycle, such as virus propagation and/or persistence.

We conducted a detailed analysis of HPV type prevalence for each histological type of cervical ADC in Japan. In usual-type ADC, the HPV positivity rate reached nearly 90%. In contrast, among other ADC subtypes, the positivity rate was significantly lower in endometrioid and minimal deviation ADC, and only HPV-negative cases were observed in serous and clear-cell ADC. Although the number of these ADC cases was small, the results are consistent with previous studies on HPV detection in histological subtypes of cervical ADC in Europe (13) and worldwide (14), and may suggest that atypical ADC is not causally related to carcinogenic HPV infection.

Regarding HPV type prevalence in cervical glandular lesions in Japan, HPV16 and HPV18 were the two predominant types exclusively detected in AIS, ASC, and usual-type ADC. The similar distribution pattern of HPV16 and HPV18 in AIS and ASC/usual-type ADC strongly supports the concept that AIS is indeed a precursor lesion for ASC and usual-type ADC. Although the high prevalence of HPV16 and HPV18 was also reported in global analyses of cervical glandular neoplasia (14), a notable deviation from global trends is the extremely low detection rate of HPV45 in cervical ADC in Japan. This low detection rate of HPV45 was also observed in cervical squamous lesions, suggesting an overall low prevalence of HPV45 infections among Japanese women.

A major limitation of this study is that vaccination history was not collected from the study participants. Thus, the HPV type distribution in this study may have been influenced by changes in HPV prevalence attributable to vaccination. In Japan, HPV vaccination started in 2009 with the bivalent vaccine, and high vaccination rates (nearly 70%) were observed among girls aged 12 to 16 years from 2010 to 2013. However, concerns about adverse events following vaccination, coupled with the government’s subsequent suspension of vaccination, led to a sharp drop in vaccination rates (by nearly 1%) (15). This suspension lasted from 2013 to 2022, keeping vaccination rates low throughout this period. On the other hand, the high vaccination cohorts born between 1994 and 1999 reached their late 20s in 2020 and may have developed CIN2/3 caused by HPV types that are not currently targeted by vaccines. Furthermore, the prevalence of HPV16/18 in CIN2/3 and invasive cervical cancer cases in Japan was shown to decrease among women in their 20s due to vaccine effectiveness (16, 17). However, when HPV type distribution was analyzed separately between the early (2012–2019) and late (2020–2025) phases of our study period, no significant differences were observed for HPV type prevalence in CIN2/3 except for a lower HPV18 prevalence between 2020 and 2025 (Supplementary Figure 2), suggesting that vaccination may have only a minimal effect on our results. We thus believe that the proportion of vaccinated individuals in our current study was extremely low, and that our results essentially reflect the prevalence of HPV types in Japan prior to the start of vaccination. Additionally, the COVID-19 pandemic appears to have had little impact on HPV type prevalence in Japan.

In summary, our analysis of the distribution of HPV types in cervical cancer/precancer patients has shown that HPV16 carries the highest risk of progression to SCC. Although HPV52 and HPV58 appear to be associated with a lower risk of cancer development, their prevalence in SCC increased among women aged 50 years and older. Among women who have received bivalent/quadrivalent vaccination, a reduction in clinical treatment for CIN2/3 caused by HPV16/18 (i.e., clinical unmasking) increases the likelihood that HPV52/58 infection will not be removed and progress to invasive cancer (18). Indeed, the Costa Rica HPV Vaccine Trial showed that women who received the bivalent vaccine had an increased absolute risk of developing CIN2/3 attributable to non-vaccine types (19). Thus, in Japan, there is a strong demand for the widespread adoption of the nonavalent vaccine, which targets HPV52/58. Finally, the presence of ADC cases unrelated to HPV serves as a reminder that HPV testing and vaccines are not a panacea for the eradication of cervical cancer.

## Data Availability

The original contributions presented in the study are included in the article/Supplementary material, further inquiries can be directed to the corresponding author.
