# Supplementary figures and images for: Prevalence and genotype distribution of human papillomavirus in cervical cancer and precancer in Japan between 2012 and 2025: a retrospective single-center study
Source: Front Public Health. 2026 Jun 12;14:1857364. doi: 10.3389/fpubh.2026.1857364 (PMC13303488; doi:10.3389/fpubh.2026.1857364)

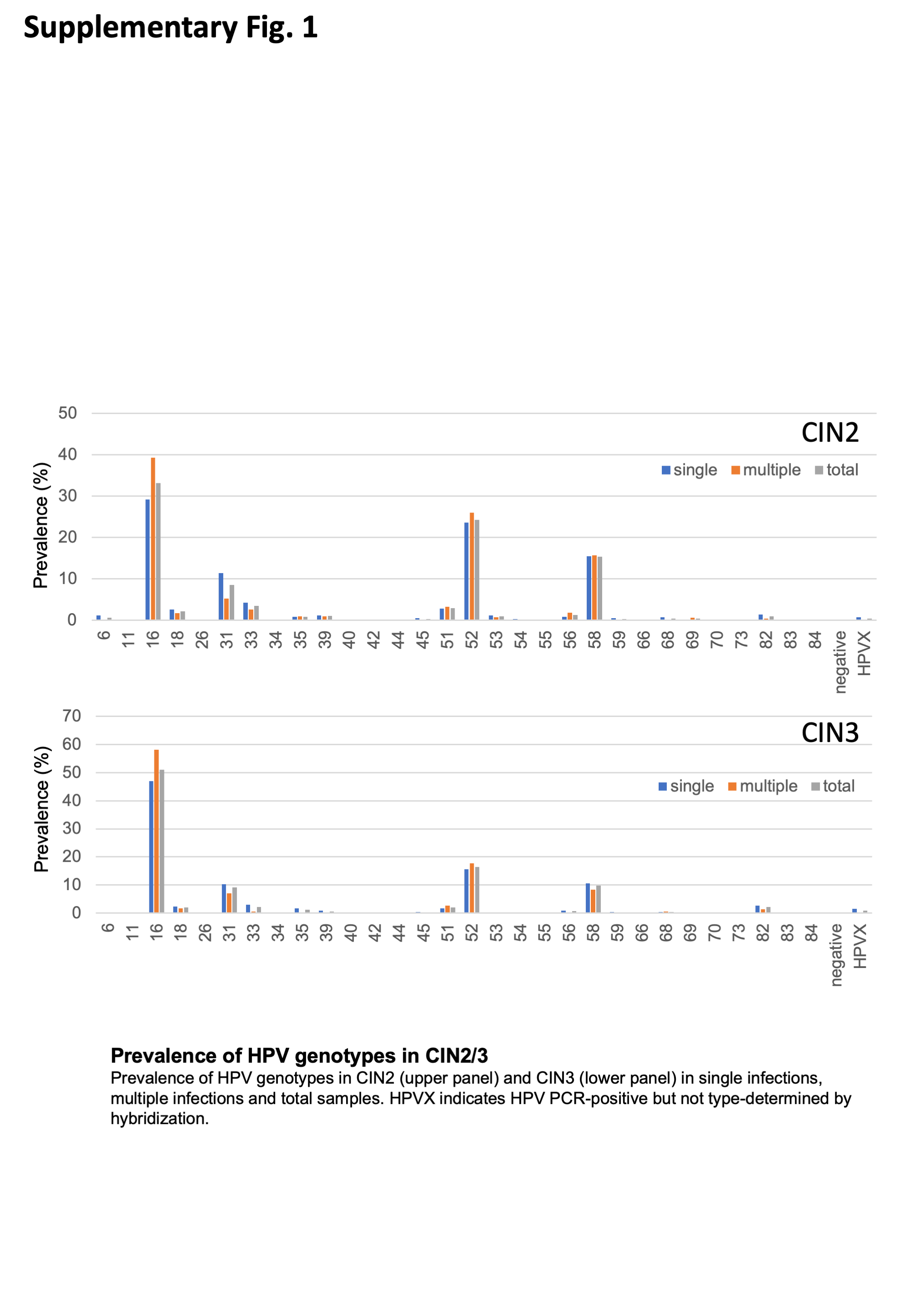

Supplement: Supplementary file 1 [file Image_1.tiff]

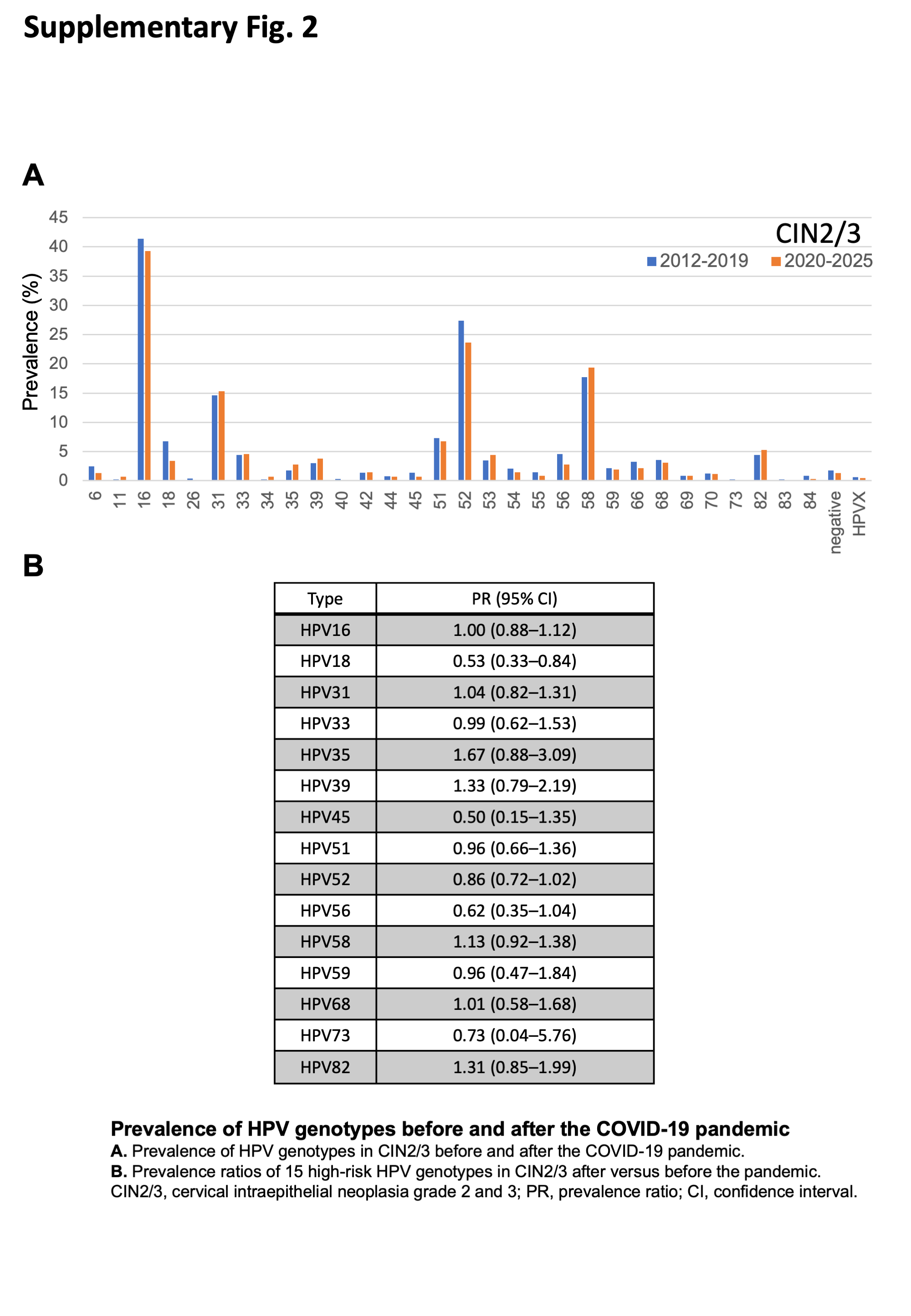

Supplement: Supplementary file 2 [file Image_2.tiff]
